# Draft Whole-Genome Sequences of Multidrug-Resistant *Escherichia coli* O157:H7 Strains Isolated from Feedlot Cattle Treated with Growth-Promoting Agents

**DOI:** 10.1128/genomeA.00284-17

**Published:** 2017-05-04

**Authors:** Muhammad A. Rehman, Catherine Carrillo, François Malouin, Moussa S. Diarra

**Affiliations:** a Guelph Research and Development Centre, Agriculture and Agri-Food Canada, Guelph, Ontario, Canada; b Canadian Food Inspection Agency, Ottawa, Ontario, Canada; c Centre d’Étude et de Valorisation en Diversité Microbienne, Département de Biologie, Faculté de Sciences, Université de Sherbrooke, Sherbrooke, Québec, Canada

## Abstract

Enterohemorrhagic *Escherichia coli* serotype O157:H7 is a major cause of foodborne outbreaks and hemolytic-uremic syndrome. Here, we report the draft genome sequences of three antibiotic-resistant *E. coli* O157:H7 strains isolated from feedlot cattle. These draft genome sequences will aid in the development of sequence-based tools for the detection of virulence and antimicrobial resistance genotypes.

## GENOME ANNOUNCEMENT

Cattle are the major reservoir of *Escherichia coli* O157:H7 (1, 2). Growth-promoting agents (GPA), including antimicrobials, are often used in cattle production to enhance growth and prevent bacterial infections. However, selective pressures exerted by the antimicrobials have been the major driving force behind the emergence and horizontal spread of antimicrobial resistance genes (ARGs) in bacteria (3), posing serious food safety and public health concerns. The three *E. coli* O157:H7 strains selected for sequencing in this study were recovered from feedlot cattle treated with GPA (monensin, trenbolone acetate-estradiol, or oxytetracycline) (4). Antimicrobial resistance was determined using an automated broth microdilution method (Sensititre CMV3AGNF plate; Trek Diagnostic Systems, Ltd.), and strains were resistant to streptomycin (Strep), sulfamethoxazole (Sult), and tetracycline (Tetr). Here, we announce the availability of the draft genome sequences of these three Strep-resistant (Strep^r^), Sult-resistant (Sult^r^), and Tetr-resistant (Tetr^r^) *E. coli* O157:H7 strains. Genomic DNA was extracted from overnight cultures grown on nutrient agar, using the DNeasy blood and tissue kit (Qiagen). Sequencing libraries were constructed using the Nextera XT DNA sample preparation kit (Illumina, Inc., San Diego, CA), and paired-end sequencing was performed on the Illumina MiSeq platform (Illumina, Inc.), using a 600-cycle MiSeq reagent kit (version 3). Sequencing reads were analyzed and quality checked using FastQC (http://www.bioinformatics.babraham.ac.uk/projects/fastqc/) and assembled *de novo* using SPAdes genome assembler version 3.9.0 (5). Contigs <1,000 bp were removed from assemblies. On average, the genome coverage, genome size, and G+C content were 70×, 5.32 Mbp, and 51.2%, respectively. The genomes were annotated with the National Center for Biotechnology Information (NCBI) Prokaryotic Genome Annotation Pipeline (PGAP) (6), identifying, on average, a total of ~5,400 coding sequences. The assembly and annotation statistics are shown in Table 1. The detection of a comprehensive set of full-length ARGs and virulence genes in the assembled genomes was performed using the bioinformatics tools ResFinder version 2.1 (7) and VirulenceFinder version 1.5 (8) available at the Center for Genomic Epidemiology website (http://www.genomicepidemiology.org). Consistent with their antibiotic susceptibility phenotypes, four ARGs (*strA, strB, tetB,* and* sul2*) associated with resistance to aminoglycoside, tetracycline, and sulfonamide were detected in all strains (Table 1). Several virulence genes were identified, including *stx*_1a_ and *stx*_2c_ (Shiga-like toxin 1 subtype a and Shiga toxin 2 [ST-2] subtype c), *eaeA* (intimin), *tir* (translocated intimin receptor),* e-hlyA* (enterohemolysin), *iss* (increased serum survival), and *iha* (iron-regulated gene homologue adhesion). These genome sequences will contribute to increases in the number and diversity of *E. coli* O157:H7 genomes publicly available for comparative analysis. Such analyses will provide unique insights and a better understanding of the antibiotic resistance mechanisms, acquisition of virulence, phenotype-genotype correlation, and evolutionary history of this pathogen.

**TABLE 1:** Characteristics of sequenced genomes

Strain	Agent(s) (4)^a^	Accession no.	Depth of coverage (×)	Assembly size (bp)	No. of genes	No. of rRNAs	No. of tRNAs	Virulence genes	Antimicrobial resistance genes
LEN02-0647-1	Rumensin	MWVK00000000	60	5,325,611	5,771	10	86	*stx1, stx2, eaeA, tir, e-hlyA, iha, iss*	*strA, strB, tetB, sul2*
LEN02-9121-1	Liquamycin + Rumensin	MWVL00000000	111	5,326,882	5,775	10	86	*stx1, stx2, eaeA, tir, e-hlyA, iha, iss*	*strA, strB, tetB, sul2*
LEN03-5497-2	Liquamycin + Revalor S	MWVM00000000	40	5,325,325	5,769	10	88	*stx1, stx2, eaeA*,* tir, e-hlyA, iha, iss*	*strA, strB, tetB, sul2*

^a^ Rumensin is the ionophore monensin. Revalor S is an implant of 120 mg of trenbolone acetate and 24 mg of estradiol-17-beta. Liquamycin is an oxytetracycline antibiotic.

### Accession number(s).

The raw reads of the DNA sequencing project have been deposited to the SRA database with the accession number SUB2427154, and the whole-genome shotgun project has been deposited at DDBJ/EMBL/GenBank under BioProject no. PRJNA273513. The genome accession numbers are listed in Table 1.
